# Using Synthetic ApoC-II Peptides and nAngptl4 Fragments to Measure Lipoprotein Lipase Activity in Radiometric and Fluorescent Assays

**DOI:** 10.3389/fcvm.2022.926631

**Published:** 2022-07-14

**Authors:** Dean Oldham, Hong Wang, Juliet Mullen, Emma Lietzke, Kayla Sprenger, Philip Reigan, Robert H. Eckel, Kimberley D. Bruce

**Affiliations:** ^1^Division of Endocrinology, Metabolism and Diabetes, Department of Medicine, University of Colorado Anschutz Medical Campus, Aurora, CO, United States; ^2^Department of Pharmaceutical Sciences, Skaggs School of Pharmacy and Pharmaceutical Sciences, University of Colorado Anschutz Medical Campus, Aurora, CO, United States; ^3^Department of Chemical Engineering, University of Colorado Boulder, Boulder, CO, United States

**Keywords:** lipoprotein lipase, hydrolysis, enzyme activity assay, lipase, microglia, post-heparin plasma

## Abstract

Lipoprotein lipase (LPL) plays a crucial role in preventing dyslipidemia by hydrolyzing triglycerides (TGs) in packaged lipoproteins. Since hypertriglyceridemia (HTG) is a major risk factor for cardiovascular disease (CVD), the leading cause of death worldwide, methods that accurately quantify the hydrolytic activity of LPL in clinical and pre-clinical samples are much needed. To date, the methods used to determine LPL activity vary considerably in their approach, in the LPL substrates used, and in the source of LPL activators and inhibitors used to quantify LPL-specific activity, rather than other lipases, e.g., hepatic lipase (HL) or endothelial lipase (EL) activity. Here, we describe methods recently optimized in our laboratory, using a synthetic ApoC-II peptide to activate LPL, and an n-terminal Angiopoietin-Like 4 fragment (nAngptl4) to inhibit LPL, presenting a cost-effective and reproducible method to measure LPL activity in human post-heparin plasma (PHP) and in LPL-enriched heparin released (HR) fractions from LPL secreting cells. We also describe a modified version of the triolein-based assay using human serum as a source of endogenous activators and inhibitors and to determine the relative abundance of circulating factors that regulate LPL activity. Finally, we describe how an ApoC-II peptide and nAngptl4 can be applied to high-throughput measurements of LPL activity using the EnzChek™ fluorescent TG analog substrate with PHP, bovine LPL, and HR LPL enriched fractions. In summary, this manuscript assesses the current methods of measuring LPL activity and makes new recommendations for measuring LPL-mediated hydrolysis in pre-clinical and clinical samples.

## Introduction

### Lipoprotein Lipase Function and Processing

Lipoprotein lipase (LPL) is a rate limiting enzyme in lipid and lipoprotein processing. LPL was initially described as “clearing factor” nearly 70 years ago, due to its ability to decrease the turbidity of lipid rich emulsions and hydrolyze neutral lipids in the presence of activating proteins (1–3). Since then, we have learned that LPL plays a critical role in lipoprotein processing throughout the body, which impacts the development of several cardiometabolic and neurodegenerative diseases. In its canonical role LPL serves as a secreted lipase that hydrolyzes triglycerides (TGs) from circulating TG-rich lipoproteins (TRLs) such as chylomicrons and very-low density lipoproteins (VLDL), making free fatty acids (FFAs) available to tissues for utilization or storage (4). Variations in the gene encoding LPL, and genes encoding factors that regulate LPL activity and processing, profoundly alter the efficiency of LPL-mediated TRL processing. The presence of such variants influences both plasma TG levels, the risk for coronary heart disease (5, 6), and the risk of Alzheimer’s disease (7). Hence, methods that can accurately quantify both circulating LPL, and the activity and abundance of LPL in a range of tissues and cells are much needed to further understand LPL biology, and to develop LPL-targeted strategies that can ameliorate cardiometabolic and neurodegenerative disease.

The human LPL gene (synonyms include *LIPD* and *HDLCQ11*) is found on chromosome 8p21.3, and contains 10 exons and 11 introns, and encodes a 475 amino acid protein. LPL is secreted in an active and glycosylated from, but secretion is dependent on several critical steps that are facilitated by key molecular chaperones and binding partners. LPL is first synthesized as a proenzyme in the rough endoplasmic reticulum (ER), where it is later glycosylated and folded in the presence of calnexin (CXN) and lipase maturation factor 1 (LMF1) (8). A specific chaperone Sel1L stabilizes the LPL-LMF1 complex and facilitates trafficking to the Golgi apparatus (9). Active LPL then enters the Golgi, where sortilin-related receptor with type-A repeats (SorLA) chaperones LPL through further post-translational modifications, sorting, and secretion, where LPL is tethered to heparan sulfate-proteoglycans (HSPG) on the cell surface (10). In the capillary lumen LPL is also bound to the glycosylphosphatidylinositol-anchored high-density lipoprotein–binding protein 1 (GPIHBP1), which anchors and stabilizes the enzyme (11). The clinical significance of LPL-processing factors is highlighted by the fact that LMF1 and GPIHBP1 genetic variants are associated with compromised LPL function and severe hypertriglyceridemia (HTG) (12).

### Lipoprotein Lipase Inhibition by Angiopoietin-Like 4

Once tethered to the cell surface or capillary lumen the activity of LPL is also dependent on the expression and availability of endogenous activators and inhibitors. For example, the Angiopoietin-Like proteins are a family of endogenous inhibitors of LPL, of which the Angiopoietin-Like 4 (Angptl4) is perhaps the best characterized. Native full-length Angptl4 (fAngptl4) is a fusion protein consisting of an N-terminal coiled-coil domain and a C-terminal fibrinogen-like domain (13) and is proteolytically cleaved by proprotein convertases (PCs) into its two isoforms with distinct functions. The N-terminal domain of Angptl4 (nAngptl4) inhibits LPL (14), whereas the C-terminus mediates antiangiogenic functions (15). Although fAngptl4 can inhibit LPL activity, proteolytic processing of fAngptl4 leads to nAngptl4 production and a greater effect on LPL activity and the ability to raise plasma TG levels (13), supporting the notion that nAngptl4 specifically inhibits LPL activity. Indeed, Angptl4-mediated inhibition of LPL activity is well established (16–20). Importantly, recent studies have shown that nAngptl4 specifically inhibits LPL activity by binding the lid domain and preventing substrate catalysis at the active site (21). For many years, the active form of LPL was thought to be a head-to-tail homodimer (22–24), and the prevailing model of Angptl4-mediated inactivation of LPL was the ability of Angptl4 to convert active LPL homodimers into readily unfolded and inactivated monomers (25). However, recent studies have shown that freshly secreted active LPL is found in a monomeric state, and that LPL-homodimer formation may be due to the presence of heparin, which is commonly used to purify or release LPL (26). In line with this paradigm shift, Angptl4 has since been shown to catalyze the unfolding of, and therefore inactivate, LPL monomers (20). Although more functional studies are needed to map the Angptl4 encounter site to LPL (20), recent Hydrogen Deuterium Exchange (HDX) Mass Spectrometry (HDX-MS) studies suggest that Angptl4 interacts with the lid (aa 89–102) and α-helix domain (aa 226–238) of LPL (21).

### Lipoprotein Lipase Activation by ApoC-II

The LPL activator ApoC-II was initially discovered in 1970 as a protein bound to VLDL and was called glutamic acid (Glu) lipoprotein or ApoLP-Glu based on its C-terminus amino acid (27). A few years later, ApoC-II deficiency was identified as the first genetic cause of HTG (28). Since then, ApoC-II variants that lead to compromised LPL function have been associated with HTG, pancreatitis, familial chylomicronemia, and gestational HTG and pancreatitis (29–32). Investigations into the regulation of the human ApoC-II gene have shown that it resides within the *APOE-APOC1-APOC4-APOC-II* gene loci on Chromosome 19 and is subject to complex tissue specific transcriptional control (33). For example, macrophage specific expression of the *APOE-APOC1-APOC4-APOC-II* locus depends on the two 620-bp macrophage-specific multi-enhancer elements termed ME.1 and ME.2 upstream of the APOC-II promoter (33, 34). Both RXR and STAT1 transcriptionally activate ApoC-II and stimulate LPL expression resulting in increased local LPL activity (34, 35). After translation, cleavage of a 22 aa signal peptide results in a 79 aa full-length protein, which does not undergo N-linked or O-linked glycosylation (33). Based on the function of known ApoC-II variants, mutagenesis studies, and investigations using synthetic ApoC-II peptides, LPL activation is mediated by the C-terminal helix of ApoC-II (36, 37). Although the precise ApoC-II-LPL interaction is unknown, it is thought that residues 63, 66, 69, and 70 bind to the lid region of LPL to facilitate TG entry into its active site (38, 39). However, considering the new crystal structures for LPL and new insights regarding the active monomeric forms of LPL, the interaction between ApoC-II and LPL, in addition to other the regulatory factors should be re-assessed.

### Existing Methods to Measure Lipoprotein Lipase Activity

The standard of care for HTG involves lifestyle, dietary (e.g., polyunsaturated fatty acid supplementation) and pharmacological (e.g., statins) interventions (40). However, these approaches are not tailored to the underlying cause of HTG. Accurately quantifying the function and activity of LPL may guide the diagnosis of LPL deficiency to highlight individuals at risk of severe and life-threatening complications such as pancreatitis, and therefore need to be followed more closely (41). While western blot analyses or ELISAs can accurately determine the abundance of the LPL protein, this does not consider the presence or absence of activating (e.g., ApoC-II and ApoA-V), inhibiting (e.g., Angptl4, ApoC-I, and ApoC-III) factors, whether LPL has been unfolded, or is in an inactive state. Therefore, the presence of the protein itself does not necessarily indicate activity. Historically, radiometric assays using ^3^H or ^14^C labeled triolein have been used to determine lipase activity in post-heparin plasma (PHP) (42, 43). These assays have been instrumental in furthering our understanding of LPL biology, however, the separation of hydrolyzed fatty acids from non-hydrolyzed trioleyl glycerol can be time consuming, low-throughput and the use of radiation is associated with health and environmental risks (44). Perhaps a greater concern, is that PHP also contains other enzymes capable of TG-hydrolysis, which could interfere with the data analysis and interpretation of radiometric lipase assays (45). Several strategies have been employed that aim to measure LPL-specific activity rather than the activity of other lipases. For example, LPL activity is very responsive to salt concentrations and high-salt conditions can inhibit LPL activity. However, high salt can also increase hepatic lipase (HL) activity; manipulating salt concentrations alone does not accurately determine LPL-specific activity (46). Therefore, assays which use LPL-specific inhibitors and activators is a more suitable approach. Here, we describe a radiometric assay to measure LPL-specific enzyme activity in PHP using a C-terminal peptide of ApoC-II, which we propose binds directly to LPL. This is a relevant admission to the protocol since this peptide is not subject to the variability associated with isolating human VLDL or serum to use as an LPL activator. In addition, in our assay we use an N-terminal peptide of the recombinant nAngptl4 protein to measure LPL activity in the presence and absence of nAngptl4, allowing us to calculate Angptl4-sensitive lipase activity, and therefore LPL-mediated hydrolysis.

Measuring LPL activity in PHP is needed to determine whether a patient or sample has compromised LPL function leading to HTG and its co-morbidities. However, we are increasingly aware that lesser degrees of HTG may result from genetic variants in one of many molecular factors that regulate LPL-mediated lipoprotein metabolism, rather than variations in the LPL gene/protein itself (29, 47–50). Hence, we have developed a modified form of the triolein-based assay that uses a patient’s preheparin serum (henceforth referred to as just serum) as an activator/inhibitor for a standardized source of LPL. For example, determining whether a patient’s serum can decrease the activity of LPL, compared to a control sample, indicates whether endogenous LPL-regulating factors are altered rather LPL being directly compromised. Although this assay does not discriminate which activator or inhibitor is affecting LPL function, it is a relatively time-efficient first-step experiment to determine that LPL regulating factors, rather than LPL, are contributing to HTG. While not the standard-of-care to date, this may guide a patient’s clinical support toward emerging strategies that specifically target LPL regulating factors, such as Angptl4 (51), ApoC-II, and ApoC-III (52, 53). Indeed, this methodology may be particularly useful for assessing lipase activity in subjects that have received treatments that specifically target ApoC-II and ApoC-III, rather than LPL (52, 53). Moreover, this experiment does not require the somewhat invasive administration of a heparin bolus to the patient and retrieval of PHP.

The PHP and serum activation/inhibition protocols described here are validated radiometric assays that allow accurate measurement of LPL activity using physiologically relevant lipid substrates, which can be tailored to answer the specific research question at hand. However, as described previously these assays are laborious, fairly low-throughput, and require the use of radiation and dedicated equipment. Therefore, for drug development studies or studies with large numbers of biological pre-clinical samples, a higher-throughput and time efficient process is needed. Therefore, here we also describe a modified fluorescent assay using a commercially available fluorogenic TG-analog, the EnzChek™ lipase substrate (54), where we also include both ApoC-II and nAngptl4 to determine LPL-specific activity. Although this has previously been demonstrated (54), here for the first time to our knowledge we also show how the EnzChek™ lipase substrate can be used to quantify LPL activity in PHP and heparin released (HR) fractions from cell types known to express and secrete LPL (BV-2 microglia). In summary, here we will provide detailed descriptions of optimized assays to determine; 1. radiometric detection of LPL activity using ApoC-II, nAngptl4 and a triolein substrate; 2. serum activation/inhibition of LPL; and 3. fluorescence detection of LPL activity using the EnzChek™ substrate. We will also make recommendations for assay selection and study design throughout, as well as highlighting caveats for each assay that may guide protocol selection for a given research question.

## Materials and Equipment

### Radiometric Detection of Lipoprotein Lipase Activity Using Triolein, ApoC-II and Angiopoietin-Like 4

#### Reagents

• 1,α-phosphatidylcholine (lecithin), Sigma P3556
• ^3^H-triolein, Perkin Elmer NET431001MC (1 mCi/mL, Specific activity: 41.6 Ci/mmol)
• ^3^H-oleic acid, Perkin Elmer NET289001MC (1 mCi/mL, Specific activity 54.6 Ci/mmol)
• Glycerol Trioleate, Sigma T7140 (500 mg)
• SCINTISAFE 30% (Fisher Scientific SX235)
• Untagged ApoC-II peptide (Genscript; Sequence: STAAMSTYTGIFTDQVLSVLKGE; untagged)
• Molecular biology grade H_2_O (Corning, 46-000-CV)
• Recombinant Human Angiopoietin-Like 4 N-Terminal Frag (R&D systems, 8249-AN-050), reconstituted in molecular biology grade H_2_O to a final concentration of 200 μg/mL
• Source of LPL [bLPL (Sigma, L2254), PHP, HR source of LPL from cells or tissues].

#### Buffers/Solutions

• Working solution of Glycerol Trioleate. Add 10 mL chloroform to yield a solution of 50 mg/mL.
• 5 M NaCl
• Tris Buffer, 2 M pH = 8.2
• 10% (FFA free) Bovine Serum Albumin (BSA) in dH_2_O, (Sigma, A-6003)
• For 50 mL no salt Krebs Ringer Phosphate (KRP) Buffer (Adjust to pH 7.4, sterile filter) (store at 4°C), 3.2 mL (1.15% KCl) (10 mM), 0.5 mL (1.52% CaCl_2_⋅2H_2_O) (1 mM), 0.16 mL (3.82% MgSO_4_⋅7H_2_O) (1 mM), 1 mL (Phosphate Buffer, 14.2 g Na_2_HPO_4_ + 20 mL 1 N HCl, dilute up to 1 L with dH_2_O, Adjust to pH 7.4) (2 mM Na_2_HPO_4_). This KRP buffer can be used to add salt to the desired concentration. We recommend (0.175 M) for optimal LPL activity.
• 100 and 1 μg/mL in 0.9% saline (Fisher Scientific, #H-19)
• Belfrage Extraction Mix (store at RT), Chloroform (683.0 mL) 34%, Methanol (770.5 mL) 39%, Heptane (546.5 mL) 27%, Bicarbonate Buffer (store at RT), Na_2_CO_3_ (2.92 g) + NaHCO_3_ (1.89 g), dilute up to 1 L, adjust pH to 10.5.

#### Equipment and Software

• Eppendorf pipettes
• Parafilm
• Dispensette dispensers
• Beckman Polyallomer Centrifuge Tubes 1 × 3 1/2″
• Fisher 13 × 100 mm glass test tubes
• Savit tube caps
• Beckman TJ6 centrifuge
• Beckman LS 600 TA scintillation counter
• Fisher HDPE 7 mL Scintillation Vial (catalog number 03-337-1)
• 1.7 mL Fisher polypropylene microcentrifuge tubes
• Fisher Scientific 550 Sonic Dismembrator
• Excel and Prism (GraphPad).

### Serum Activation/Inhibition of Lipoprotein Lipase

#### Reagents

• See section “Reagents” in Radiometric Detection of Lipoprotein Lipase Activity Using Triolein, ApoC-II, and Angiopoietin-Like 4, with the addition of preheparin human serum.

#### Buffers/Solutions

• See section “Buffers/Solutions” in Radiometric Detection of Lipoprotein Lipase Activity Using Triolein, ApoC-II, and Angiopoietin-Like 4.

#### Equipment and Software

• See section “Equipment and Software” in Radiometric Detection of Lipoprotein Lipase Activity Using Triolein, ApoC-II, and Angiopoietin-Like 4.

### Fluorescence Detection Lipoprotein Lipase Activity Using the EnzChek™ Substrate

#### Reagents

• EnzChek™ Lipase Substrate, green fluorescent, 505/51 CAT: E33955
• Heparin Sodium (Fisher #BP2425)
• DMSO
• Zwittergent^®^ 3-12 Detergent CAT: 693015
• Molecular Grade Methanol
• 1 M NaCl
• 1 M Tris–HCl pH 8.0.

#### Buffers/Solutions

• 10 mg/ml Heparin Sodium stock (aliquot in 50 μL, store at −20°C)
• Heparin containing KRP (25 μL 10 mg/ml Heparin Sodium, and 4.975 μL KRP)
• Dissolve EnzChek in 20% DMSO to a final molarity of 100 μM
• Dissolve Zwittergent with a final concentration of 8% Zwittergent in molecular grade H_2_O and molecular grade 1% Methanol
• 4× assay Buffer (600 mM NaCl, 80 mM Tris, molecular grade H_2_O).

#### Equipment and Analysis Software

• 96-well black-walled plate for fluorescence
• Plate reader capable of temperature control and fluorescent detection
• Graph pad (or other software capable of fitting a Michaelis–Menten curve).

## Methods

### Radiometric Detection of Lipoprotein Lipase Activity Using Triolein, ApoC-II, and Angiopoietin-Like 4

**FIGURE 1:**
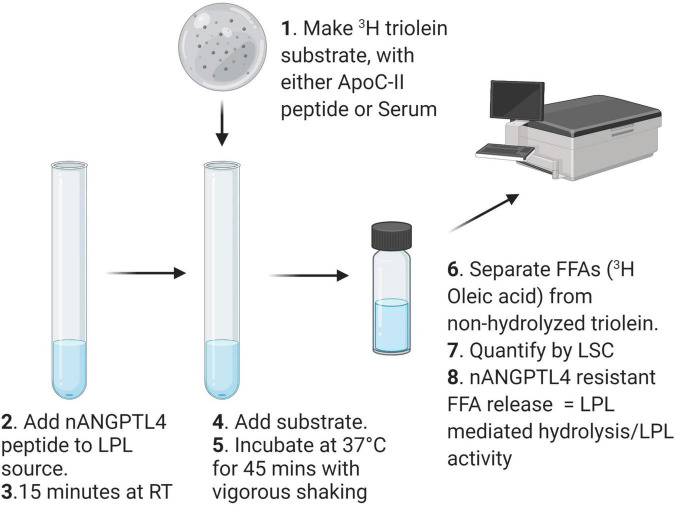
Schematic workflow for measuring LPL activity with a triolein substrate.

Radiometric detection of LPL activity is an appropriate method for determining the activity of heparin-released LPL activity in PHP from humans or from pre-clinical animal models. Importantly, the protocol, can be modified to answer a specific research question e.g., changes to the substrate and PHP dilution or treatment can easily be implemented. A schematic describing the workflow of the method detailed in this section is shown in Figure 1.

#### Method

##### Make the Organic Portion of the Triolein Substrate

In a Beckman Polyallomer Centrifuge Tube mix together 20 μL ^3^H-triolein (1 mCi/mL), 200 μL Glycerol Triolate (50 mg/mL), and 20 μL lecithin (12.5 mg/mL). Dry mixture under N_2_ for 10 min, or until no liquid visibly moves when the tube is rotated.

##### Make the Aqueous Portion of the Triolein Substate

Add 1.9 ml dH_2_O, 1 mL Tris (2 M, pH = 8.2), 0.8 ml 10% BSA, ApoC-II synthetic peptide (5–10 μM).

##### Sonicate the Mixture to Make an Emulsion

Ensure the part of the tube containing the substrate is submerged within an ice-slush bath. Insert sonicator probe 1–2 mm into the substrate mixture. Pulse-sonicate [output frequency: 20 kHz, 10 s on, 10 s off, (duty cycle = 0.05%) for 32 cycles]. The clear mixture should now be an opaque emulsion. Cover with parafilm and place on ice until ready to add to LPL source. Although the substrate should be made fresh the day of the assay, the substrate can stay on ice for several hours while preparing samples. However, ensure to gently mix the substrate prior to adding to the source of LPL.

##### Prepare Lipoprotein Lipase Samples

For human PHP samples we recommend 1:100 dilution with KRP (0.175 M NaCl). For different samples that have not been tested previously a concentration range optimization experiment may be appropriate. Keep the samples on ice while preparing the assay, and work quickly once the samples are diluted. Once all samples are diluted, add 100 μL of each sample (in triplicate) to a glass test tube. Prepare two sets of samples,

##### Angiopoietin-Like 4 Inhibition

Add Angptl4 to each “+Angptl4” sample, at 10 μg/ml, and incubate at RT for 15 min. Add an equal volume of vehicle (molecular grade H_2_O), to the remaining samples. Due to the effect on LPL activity, ensure all + and − Angptl4 samples are incubated at RT for the same amount of time.

##### Prepare Internal Standards

For each assay a number of internal standards are required in order to control for variability in the substrate generation (substrate dose), oleic acid dilution [oleic acid dose fatty acid recovery (oleic acid recovery)], KRP buffer composition (KRP Blank), as well as measurement of a known source of LPL that can be used in each assay and is consistent across a given research project [quality control (QC)]. To prepare a final volume of 100 μL for each standard:

• **Substrate dose** = 100 μL substrate into 5 ml Scintillation fluid. Does not require processing through the hydrolysis reaction but should be processed at the same time to account for degradation.
• **Oleic Acid dose** = 20 μL ^14^C-Oleic acid into 5 ml Scintillation fluid. Does not require processing through the hydrolysis reaction but should be processed at the same time to account for degradation.
• **Blank** = 100 μL KRP.
• **Oleic acid recovery dose** = ^14^C-Oleic acid (20 μL 1 mCi/mL) + KRP (80 μL).
• **Bovine LPL QC** = 100 μL 1:1,000 dilution (in KRP) of bovine LPL (Sigma L2254, 1 unit per μL).
• **PHP control QC** = 100 μL 1:100 dilution of PHP from typical patient or pooled sample from typical and healthy population (in KRP).

##### Hydrolysis Reaction

Add 100 μL of substrate to each sample and standard. Incubate all tubes in a shaking water bath at 37°C for 45 min exactly. To terminate the reaction, add 3.4 mL of Belfrage to all samples, followed by 0.96 mL of bicarbonate buffer to each tube. Ensure all tubes are carefully capped and shake vigorously for 5 min.

##### Phase Separation

To separate the hydrolyzed FAs from non-hydrolyzed triolein centrifuge at 4°C for 20 min × 3,000 rpm (Beckman, TJ6). When removing and handling samples after centrifugation be careful not to disturb the interface. Transfer 500 μL of the top aqueous phase to a scintillation vial containing 5 ml SCINTISAFE. Vortex each sample for 10 s, and then read all samples in a scintillation counter capable of measuring ^3^H for 2 min, providing ^3^H counts per minute (CPMs) for each sample.

#### Data Analysis

1. Subtract the blank CPMs from each sample CPM.
2. Determine total lipase activity for each sample using the following formula:

$$Activity\left(nmol\text{FFA}/mL/min\right)=\frac{\mathrm{Sample}\mathrm{CPM}\times K\times 10}{DOSEcounts\times Recovery}$$

Where K equals


$$\begin{array}{c} K=\frac{mLupperphase}{mLcounted}\times nEqTGoleicacid/tube\\ \;\times \frac{dilution}{inc.time}\times 100 \end{array}$$

$$\begin{array}{c} nEqTGoleic=\frac{50\frac{mg}{ml}\times \frac{0.2}{4.0}}{885.5g}\times 0.100mL\times 3oleicacidpermole\\ \;\times 10^{6}=423 \end{array}$$

$$\frac{2.45mLupperphase}{0.5mLcounted}\times 100=490$$

$$Recovery=\frac{OARScounts}{OADcounts}\times 490$$

1. To calculate LPL-specific activity subtract +Angptl4 lipase activity (Angptl4 resistant lipase) away from total lipase activity. Express data as nmol FFA/mL/min. Transform to other units (e.g., per hour) if required.

### Assessing Ability of Preheparin Serum to Inhibit or Activate Lipoprotein Lipase

In many instances, HTG is not caused by LPL variants leading to compromised LPL protein structure and function, but by a change in the endogenous circulating factors that are necessary to maintain LPL activity in each state. As a first-step approach to determine whether reduced lipase activity is a product of endogenous inhibition we have developed the following assay. The method outlined above is almost identical to the method described in section “Radiometric Detection of Lipoprotein Lipase Activity Using Triolein, ApoC-II, and Angiopoietin-Like 4,” therefore, here we only detail the steps that differ from the above protocol.

#### Method

##### Make the Aqueous Portion of the Triolein Substate

Add 1.9 ml dH_2_O, 1 mL Tris (2 M, pH = 8.2), 0.8 mL 10% BSA, 0.3 mL preheparin serum (substitute for ApoC-II).

##### Prepare Lipoprotein Lipase Samples

To ensure that the serum substrate is the only variable for these assays we suggest using a constant and known source of LPL (not patients PH), such as bovine LPL (bLPL) (Sigma). A working concentration of 1:1,000, bLPL is sufficient to determine differences in serum mediated activation/inhibition of LPL-mediate hydrolysis.

#### Data Analysis

Determine LPL activity using the same standards and formulas shown above. Importantly, for each substrate a complete set of standards should also be generated for accurate determination of LPL activity, e.g., substrate dose, blank, oleic acid recovery dose. For clinical research studies, comparison against a typical healthy patient sample, or patient sample pool should be considered. For drug intervention studies over time, ability for a patient’s serum to increase.

### Fluorescence Detection of Lipoprotein Lipase Activity Using the EnzChek™ Substrate

**FIGURE 2:**
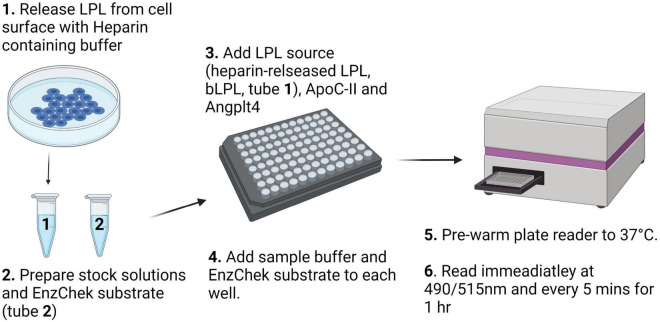
Schematic description of the workflow for the fluorescence detection of LPL activity using the EnzChek™ substrate.

Fluorescent detection of LPL activity presents a 96-well plate assay format and relatively high-throughput alternative to the measuring LPL activity using a range of LPL sources where biological and technical replicates are too high to perform the triolein-substrate assay, or in laboratories where studies using radioactive materials are prohibited. A schematic workflow for this protocol is shown in Figure 2.

#### Method

##### Heparin Released LPL From Surface of Heparin Secreting Cells

Grow LPL secreting cells to confluency. Aspirate media and wash cells 3× with room temperature sterile PBS. Aspirate PBS. Leave plate tilted for 1 min to ensure that remaining buffer pools, can be aspirated, and will not dilute the LPL-enriched HR fraction. Add heparin–containing KRP buffer (300 μL per confluent 10 cm^2^ plate) for 5 min, then immediately place on ice before quantification of lipase activity. A minimum volume of 100 μL HR per well is needed for EnzChek™ assay.

*Prepare bLPL stock*. Dilute bLPL in phenol free DMEM media without FBS 1:500.

*Prepare PHP stock*. A final dilution of 40× was needed to read LPL activity in PHP. Higher concentrations appeared to be affected by the natural yellow color of PHP resulting in inconclusive, or “noisy,” readings. Therefore, for each well combine 5 μL of the PHP sample with 95 μL molecular grade H_2_O for each well per condition.

*Prepare Plate*. Four conditions/controls were tested per LPL source at equal volumes: Blank, LPL, LPL + ApoC-II, and LPL + Angptl4 in quadruplicate. ApoC-II was added at a concentration of 1 μM to increase LPL activity. Angptl4 was added at 2.7 μg/mL (for a final dose of 200 ng/well) to decrease LPL activity in each LPL source (i.e., HR, bLPL, or PHP) and incubated for 15 min at RT along with a control. During the 15-min incubation aliquot 100 μL of each sample into each well. Add molecular grade H_2_O to each well to a final volume of 150 μL. bLPL could be read at a smaller volume with just 75 μL bLPL with no added H_2_O in each well.

*Pre-warm fluorescent plate reader to 37°C*.

*Prepare EnzChek™ Stock solutions*. A Final EnzChek™ concentration of 0.625 μM is necessary. While the plate reader is warming, prepare 80× EnzChek™/Zwittergent by mixing EnzChek and Zwittergent in equal parts (50 μM EnzChek and 4% Zwittergent). Dilute the 50 μM EnzChek™/Zwittergent stock in 4× Sample buffer 1:20 for a 200 μL well volume or 1:40 for a 100 μL well volume. Immediately before read, add the EnzChek™ stock to each well to dilute 4× buffer to 1×.

*Read Plate*. Read the plate immediately at 490/515 excitation/emission at 37°C every 5 min for 1 h.

#### Data Analysis

A blank is comprised of the 4× buffer, diluted to 1× in each well, DMEM, H_2_O, and EnzChek/Zwittergent spike without any LPL. The average blank value is subtracted from each well per timepoint, producing a blank subtracted data set. Each technical replicate was then divided by the average fluorescent value of the first time point of the control group to produce a fold change from baseline. All wells in each condition are then averaged and the standard error calculated in GraphPad Prism V8. These values are then plotted, and a Michaelis–Menten line is fitted to each curve. The proper use of the Michaelis–Menten fit is tested as the best model by GraphPad. Differences are calculated by two-way ANOVA at each individual time point of Angptl4 and ApoC-II treated compared to Control.

## Results

### Salt Concentration of Working Buffer Is Important for Accurately Measuring Lipoprotein Lipase Activity in Postheparin Plasma

**FIGURE 3:**
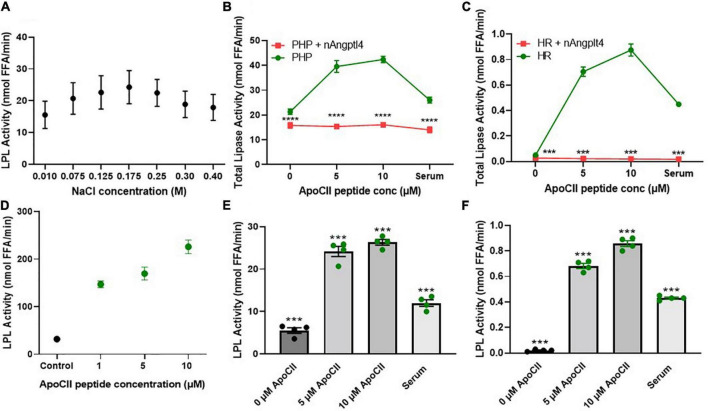
NaCl, inhibitor (nAngptl4) and activator (ApoC-II peptide) standardization of the triolein substate method of measuring LPL activity. **(A)** Optimization of NaCl concentrations within KRP buffer used to dilute PHP (1:100) isolated from pool of healthy subjects. **(B)** Total lipase activity in PHP (1:100 dilution in 0.175 M NaCl KRP) + and – 5 μg/mL nAngptl4 in presence of 0, 5, or 10 μM ApoC-II peptide. **(C)** Total lipase activity in HR + and – 5 μg/ml nAngptl4 in presence of 0, 5, or 10 μM ApoC-II peptide. **(D)** Activation of bLPL with increasing ApoC-II peptide concentration. **(E)** LPL activity in PHP with increasing concentrations of ApoC-II peptide compared to serum as a source of ApoC-II. **(F)** LPL activity in KH with increasing concentrations of ApoC-II peptide compared to serum as a source of ApoC-II. ***P < 0.001; ****P < 0.0001.

Historically, LPL activity has been shown to be sensitive to the concentration of NaCl in the working buffer solution. Previous assays have reported the use of “low salt” potassium phosphate buffers either as a component of the lipid substrates or as a diluent for the LPL source (e.g., PHP) to measure LPL activity where the NaCl concentration ranges from 0.1 to 0.3 M NaCl (46, 55–57). To determine the optimal NaCl concentration to use as a diluent buffer for the triolein-substrate assay, we diluted PHP taken from four “typical” healthy subjects in KRP buffer containing 0.01–0.4 M NaCl and measured LPL activity using the triolein substrate assay described in “Radiometric Detection of Lipoprotein Lipase Activity Using Triolein, ApoC-II, and Angiopoietin-Like 4.” We found that LPL activity was highest when PHP was diluted in 0.175 M NaCl (Figure 3A), and therefore we recommend using 0.175 M NaCl when measuring LPL activity.

### N-Terminal Angiopoietin-Like 4 Peptide Robustly Inhibits Lipoprotein Lipase Activity in Triolein-Based Assays

Previous reports have taken advantage of the salt-sensitivity of LPL by using “high salt” concentrations to specifically inhibit LPL activity in PHP. Although both HL and LPL are the major lipases in human PHP, in contrast to LPL, HL activity is increased in the presence of high salt and does not require further activation by circulating serum-derived factors (58). Therefore, HL activity has previously been calculated by inhibiting LPL activity with 1 M NaCl and measuring the salt-resistant lipase activity (59). However, 1 M NaCl only partially inhibits LPL activity in PHP from HL-deficient mice, suggesting that “high salt” is not an optimal method of LPL inhibition (46). Immunochemical inhibition of HL using HL-specific antibodies partially resolves this issue (55, 56, 60, 61). However, all forms of immunoassays are dependent on variations in antibody titer and specificity, whether the antibody was raised against a polypeptide or protein, or even between batches of the same antibody. Since the N-terminal domain of Angptl4 (nAngptl4) is a reversible, noncompetitive inhibitor of LPL (62), we established whether nAngptl4 would be a suitable alternative mode of LPL inhibition in triolein-substrate assays. Using a method initially adapted from Basu et al. (54), we established that 10 μg/mL nAngptl4 peptide could specifically inhibit LPL activity PHP (Figure 3B), and in LPL-enriched HR fractions isolated from cell lines that abundantly express and secrete LPL (BV-2 murine microglia) (Figure 3C), even in the presence of synthetic ApoC-II peptide or human serum as a source of ApoC-II. Since the circulating levels of Angptl4 have been reported to range from 455 to 961 ng/mL in patients with varying degrees of atherosclerosis (63). Hence, 10 μg/mL is sufficient to saturate LPL activity when LPL is within the typical range. However, in severe HTG or other non-representative samples a range of 5–20 μg/mL may be considered. As expected, in our study nAngptl4-mediated inhibition of LPL activity only depleted half of the total lipase activity in typical human PHP, with the nAngptl4-resistant lipase activity most likely being HL, and to a much lesser extent endothelial lipase (EL) (Figure 3B). Interestingly, all lipase activity in the HR LPL-enriched fractions taken from LPL-producing BV-2 murine microglia cells was lost following nAngptl4 supplementation, suggesting that LPL accounts for all lipases present on the cell surface of microglia (Figure 3C). Overall, we recommend the use of nAngptl4 in all assays measuring LPL activity.

### ApoC-II Peptide Robustly Activates Lipoprotein Lipase Activity in Triolein-Based Assays

Like the methods used to specifically inhibit LPL activity, the methods used to activate LPL vary considerably across protocols. While almost all assays rely on ApoC-II mediated activation of LPL, the source of ApoC-II can range from serum samples taken from individuals or a pool of healthy individuals (46, 55, 56, 64), VLDL (65), ApoC-II protein (66), or ApoC-II peptide (27), leading to variable substrate composition and inconsistent results. In response, we have synthesized an ApoC-II peptide fragment corresponding to amino acids 55–78, and tested its ability to activate LPL in PHP, HR LPL from microglia, and bLPL (Figures 3B–D). Previous studies have shown that this ApoC-II peptide is just as potent as the full length ApoC-II protein at activating LPL (27). Here, we show that a final concentration of 5 μM ApoC-II can robustly increase LPL activity in PHP, HR fractions, and bLPL (Figures 3B–F). Unexpectedly, substrates made with 0 ApoC-II were able to minimally activate LPL within PHP from a pool of typical subjects (Figure 3B). This suggests that the ApoC-II concentrations within a concentrated PHP sample is sufficient to activate LPL. In support of this, samples with no endogenous ApoC-II (HR LPL fractions from microglia), showed no LPL activity when serum or ApoC-II was absent from the substrate, yet showed a significant increase in LPL activity with addition of ApoC-II or serum to the substrate (Figure 3C). Overall, these findings corroborate the fact that circulating concentrations of ApoC-II are approximately 4.5 μM (67). However, considering ApoC-II is a component of VLDL and can be increased in patients with HTG, we recommend using 5 μM ApoC-II peptide 55–78 as an LPL activator in triolein-substrate LPL activity assays, but would also recommend testing with 10 μM in samples from patients or pre-clinical samples with increased TGs.

### Using Preheparin Serum to Activate Lipoprotein Lipase Can Determine the Presence of Endogenous Activators and Inhibitors

**FIGURE 4:**
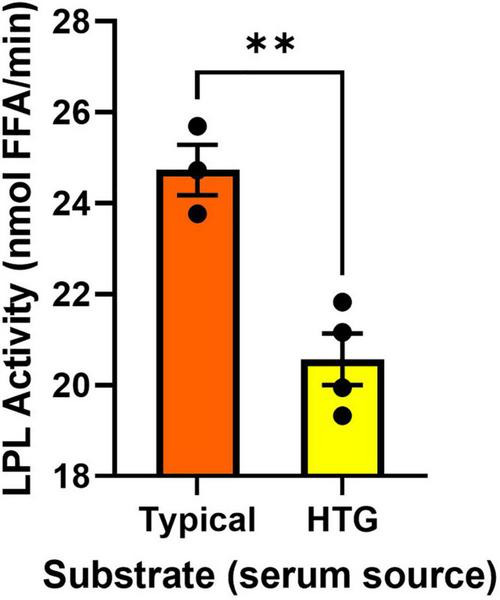
Serum activation/inhibition of a known LPL source. **P < 0.01.

Previous reports have suggested that the utility of measuring lipase activity in PHP is limited, since low LPL activity (68) is not always related to LPL polymorphisms (60). In addition, studies using triolein substrates and preheparin serum as a source of ApoC-II have also shown poor relationships between the activity of PHP lipases and the absolute turnover of serum TGs (61). Although these studies have reported important outcomes, it is also plausible that these inconsistencies stem from caveats in the experimental approach. For example, HTG can be caused by genetic variation in an array of molecular factors that enhance or inhibit LPL activity (29, 47–50). Therefore, in an individual with HTG the structure and function of the LPL enzyme may be uncompromised but, for example, an LPL activator such as ApoC-II may be compromised. In such a case, examining LPL activity in PHP using serum from a healthy/typical donor would not be able to resolve altered endogenous inhibition of LPL. To counter this problem, we designed an assay where serum from an individual is used as a source of circulating endogenous activators and inhibitors. Specifically, the serum is included as a component of the triolein-containing substrate emulsion. This substrate is added to a known and well characterized source of LPL. Here, we used bLPL, since no other LPL-regulating factors are present, but this assay could also be performed with HR LPL, or recombinant commercially available human LPL. The presence of LPL-regulating factors was evaluated by comparing LPL activation when the substrates contained serum from a typical healthy sample pool, versus LPL activation when the substrates contained serum from individuals with known HTG (Figure 4). We found serum from four individuals with HTG was less able to activate bLPL compared to serum from a typical patient pool (Figure 4). We would recommend employing this study design when LPL-independent mechanisms of HTG are suspected, or if PHP lipase activity appear normal despite HTG. In addition, this approach would be an appropriate study design when testing anti-hyperlipidemic drugs when a serum from the same individual can be compared over time.

### ApoC-II Activation and Angiopoietin-Like 4 Inhibition Can Accurately Determine Lipoprotein Lipase Activity Using the EnzChek™ Substrate in Post-heparin Plasma, Heparin Released Lipoprotein Lipase, and Bovine Lipoprotein Lipase

**FIGURE 5:**
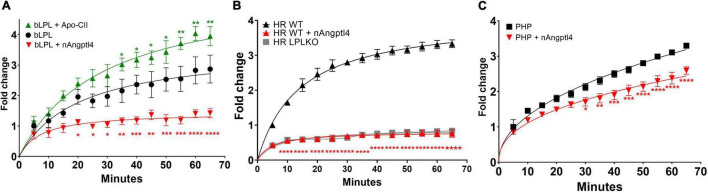
Fluorescence detection of LPL activity using the EnzChek™ substrate, ApoC-II activation and nAngptl4 inhibition to determine LPL activity using **(A)** bLPL **(B)** heparin-released (HR) LPL from LPL secreting BV-2 microglia, and LPL knock out (LPL KO) cells as a control, and **(C)** PHP as a source of LPL. *P < 0.05; **P < 0.01; ***P < 0.001; ****P < 0.0001.

Although determining LPL activity using physiologically relevant triolein substrates with ApoC-II and nAngptl4 provides an accurate and gold-standard approach for measuring lipase activity, particularly in PHP, the methodology remains cumbersome, lengthy, and reliant on radiolabeled lipids that are a health and environmental hazard. Therefore, several research groups have aimed to develop novel TG-like substrates that can be used in a high-throughput 96-well assay format, does not require the use of radioisotopes, nor complex procedures to separate hydrolyzed from non-hydrolyzed substrate (54, 69, 70). While these assays are cost-effective and efficient, particularly when measuring LPL activity in pre-clinical samples, in samples derived from cultured cells, or in drug-development screens, they have not been evaluated with PHP samples. Here we have shown that the EnzChek™ substrate can be used to quantify LPL activity in bLPL, (Figure 5A), LPL enriched HR fractions from BV-2 microglia, but not in HF fractions from BV-2 microglia where LPL expression has been knocked out using CRISPR-Cas9 (Figure 5B), and PHP (Figure 5C). Several optimization experiments were needed to accurately and reproducibly measure LPL activity in PHP, with optimal activity being observed at a dilution of 2:0.4 (EnzChek™ reagent and buffer:PHP). Moreover, we have shown that LPL-mediated hydrolysis of the EnzChek™ substrate can be robustly inhibited by 5 μg/ml nAngptl4 and increased by 10 μM ApoC-II peptide using bLPL as a source of LPL (Figure 5A). Interestingly ApoC-II-mediated activation did not increase LPL activity in HR or PHP, which may reflect the structure of the TG analog substrate, the relative composition of the biological sample, and the presence of endogenous ApoC-II (data not shown). However, hydrolysis of the EnzChek™ substrate was inhibited by the n-terminal Angptl4 fragment in HR and PHP. Therefore, we recommend inclusion of the ApoC-II and nAngptl4 peptide in the experimental design of studies aiming to the measure LPL activation using the EnzChek™ assay.

## Discussion

While there are several methods to determine LPL activity, each has several caveats precluding its applicability to a given research, or diagnostic objective. Here, we describe, evaluate, and optimize methods to; (1) measure LPL activity in PHP; (2) determine presence of circulating factors that may inhibit or activate LPL; and (3) to determine LPL activity in PHP and heparin-released LPL-enriched fractions using the EnzChek fluorescent substrate. We have made a number of changes to existing protocols, most notably, the use of defined salt concentrations (0.175 M NaCl) in the working buffer, the use of an ApoC-II peptide as a potent, readily available and cost-effective LPL activator, and the use of the nAngptl4 peptide as an LPL-specific inhibitor. Here, we describe these methods in detail and recommend the use of ApoC-II peptides and nAngptl4 to reduce variation and increase reproducibility when quantifying LPL activity in a range of samples.

In our analysis we observe a robust reduction in total lipase activity following supplementation with the nAngptl4 fragment. There is considerable data to support the notion that Angptl4 regulates LPL activity at a post-translational level (16–20). Indeed, recent studies have shown that nAngptl4 specifically inhibits LPL activity by binding the lid domain and preventing substrate catalysis at the active site (21). Since the reciprocal studies have not been performed to assess whether nAngptl4 can inhibit the activity of other lipases such as HL, and to a lesser extent EL, that may be present in PHP, we cannot rule out the possibility that nAngptl4 may reduce total lipase activity. However, in recent studies using Angptl4 knockout mice, no effects on HL or total lipase were observed, whereas LPL activity was modified following Angptl4 depletion (71). In addition, in the present study, we show that nAngptl4 treatment completely abolishes lipase activity in extracellular fractions from microglia that only express LPL, whereas nAngptl4 only partially inhibits lipase activity in PHP, suggesting that HL, the second most abundant in PHP, is at least partially resistant to nAngptl4-mediated inhibition. Additionally, EL is mostly a phospholipase and has very little TG lipolytic activity (44). Further studies that directly assess the ability of nAngptl4 to inhibit HL and EL, while beyond the scope of this manuscript, should be considered going forward. Nonetheless, the addition of both ApoC-II and Angptl4 in our study design provides an additional layer of control when measuring LPL-specific catalysis of triolein.

While the triolein-substrate method for quantifying LPL activity is fairly well established, there are variations in its execution from laboratory to laboratory, that require consideration when interpreting the results. For example, while almost all assays rely on ApoC-II mediated activation of LPL, the source of ApoC-II can range from serum samples taken from individuals or a pool of healthy individuals (46, 55, 56, 64), VLDL (65), ApoC-II protein (66), or ApoC-II peptide (27). However, using human serum as a source of ApoC-II is subject to considerable variability, between individuals, and even within the same individual over time, nutritional status, and other exposures that may affect TG and ApoC-II levels. Although using a pooled sample of human preheparin serum may prevent variation within a single testing laboratory, different laboratories across the world generate their own reference pool of healthy human serum, which is subject to regional variability and may not be replicated by other research teams. Using VLDL prepared by ultracentrifugation as a source of TG and ApoC-II is advantageous, but harsh ultracentrifugation procedures can non-discriminately de-proteinate lipoproteins leading to variation in substrate composition. Using recombinant and purified sources of ApoC-II may limit variability, but recombinant apoproteins are very expensive and the quality may vary depending on the expression system and purification procedure. To counter these issues, here we present the use of a synthetic ApoC-II peptide, which is a potent LPL activator, is not subject to biological variation, is readily available, and is relatively cost-effective. Notably, in recent studies other ApoC-II peptides have been used to mimic the ApoC-II activation of LPL while also blocking the LPL-inhibiting effect of ApoC-III and lower plasma TG (53). Therefore, the peptides described in the study by Wolska et al. (53), could easily be incorporated into the experimental approaches described in this manuscript as an additional measure of LPL-specific activity. Overall, we recommend the simultaneous use of an ApoC-II peptide, in addition to the nAngptl4 peptide for a reproducible method of determining LPL-specific lipase activity in a range of biological samples.

However, it is important to note that even following our recommendations, the ^3^H-triolein-based assay still requires specialized equipment and accommodations for using radioactive isotopes and remains low-throughput compared to the 96-well plate format colorimetric and fluorometric methods of detecting LPL activity. Hence, we would recommend making these changes to an existing protocol or reserving this method for clinical or pre-clinical evaluation of LPL activity.

**FIGURE 6:**
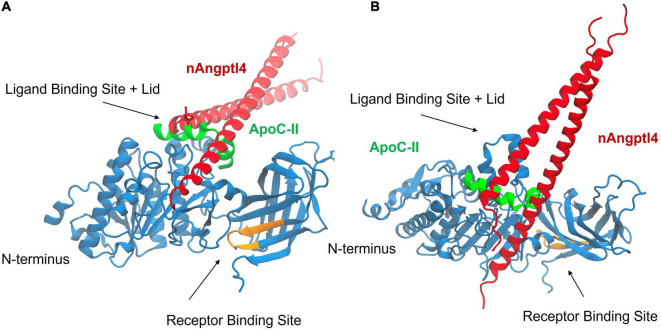
Results from molecular docking of LPL (blue) with the synthetic ApoC-II peptide (green), and nAngptl4 fragment (red) used in the experimental protocol, showing the top HDOCK-scored complex from both a front view **(A)** and top view **(B)**. The receptor binding site of LPL is highlighted in orange. The results show that the ApoC-II peptide and nAngptl4 fragments are expected to bind to the ligand binding site and lid domain of LPL, consistent with our experimental data.

Drug development studies have utilized Angptl4 to determine whether small molecule LPL activators can enhance LPL activity and reverse Angptl4-mediated inhibition (72). Hence, how nAngptl4 and ApoC-II peptides interact with LPL is of major interest to researchers pursuing LPL-focused drug development studies, to elucidate the mechanism-of-action of potential drugs, and to determine the utility of this specific assay design for their studies. Here, we used HDOCK to perform rigid-body docking calculations to predict the preferred binding interactions between the ApoC-II peptide (aa 56–78) and the N-terminal Angptl4 fragment with LPL (73). The visualization software Visual Molecular Dynamics (VMD) was used to analyze the results from the docking calculations (74). As input to HDOCK, we used the AlphaFold structure of human LPL, which closely resembles recently elucidated crystal structures of LPL, but also includes predicted coordinates for missing amino acid residues that were not resolved by crystallography (75). The AlphaFold structures for LPL (76), ApoC-II (77), and Angptl4 (78) were modified prior to docking so that they more closely represented the structures used in the experiments (i.e., LPL without the signal peptide, the region of ApoC-II covered by the synthetic peptide only, and the N-terminal fragment of Angptl4, respectively). Separate HDOCK calculations were performed with the ApoC-II peptide and LPL, and with Angptl4 and LPL. Figure 6 shows VMD snapshots of the overlay of the top HDOCK-scored complexes for the two separate calculations. The results show that both the ApoC-II peptide and the N-terminal Angptl4 fragment preferentially bind to the ligand binding site and lid domain of LPL. Furthermore, the preferred interactions predicted by HDOCK are consistent with previous studies using full-length ApoC-II and Angptl4 (27, 38, 39). This suggests that both the ApoC-II peptide and nAngptl4 fragment interact with LPL like the full-length proteins, supporting their functionality in the LPL assays described above. Moreover, this highlights the utility of this assay as a physiologically relevant method in drug development studies.

While triolein assays are often used to determine whether a patient may have LPL deficiently leading to HTG, many patients do not carry an LPL variant leading to compromised LPL activity, and LPL activity can appear normal in the cell-free triolein substrate assay described above. For these patients, an alternative methodology can be adopted to assess the presence and relative abundance of circulating endogenous factors that may inhibit LPL activity, leading to HTG. Here, we also present a modified version of the triolein-based substrate assay to answer specific research questions pertaining to the relative abundance of circulating factors that may inhibit LPL. Although this assay will determine whether activating/inhibiting factors are present in each sample the assay will not indicate which molecular factors are dysregulated to alter LPL activity. In addition, outcomes from this assay may be challenging to interpret, since in patients with HTG, LPL-regulating factors may fall or rise to compensate for the dysregulated or variant factor. Nonetheless, this assay will indicate whether LPL-regulating factors, rather than LPL enzymatic activity, is compromised and will generate pre-requisite data to necessitate further characterization of LPL-regulating factors.

Importantly, methods to assess LPL activity have been instrumental in the discovery of LPL structure and function, and its preferred substrate(s). For example, it is often forgotten that although the canonical role of LPL is the hydrolysis of TGs within TRLs, LPL also has phospholipase A_1_ activity (79, 80), and can hydrolyze lipoprotein phospholipids, and sonicated phosphatidylcholine vesicles when activated by ApoC-II (81). Using isolated lipoproteins to interrogate LPL biology can be challenging due to the complexity and heterogeneity of the lipoprotein particle, in addition to the relative abundance of activating and inhibiting factors that may confound interpretation. In response, non-lipoprotein substrates have been developed to further interrogate LPL function. *P*-nitrophenyl butyrate (PNBP) was initially developed as a short-chain fatty acid (SCFA) substrate for LPL (82), based on the fact that LPL can also catalyze the hydrolysis of SCFA esters such as tributyrin, and, *p*-nitrophenyl acatate in the absence of ApoC-II (83, 84). Although the colorimetric change that occurs following LPL-mediated hydrolysis of PNBP provides a convenient way of assessing LPL-mediated hydrolysis, using PNBP as a substrate to measure LPL activity in biological samples is not physiologically relevant since PNBP prevents LPL-mediated TG hydrolysis, and the hydrolysis of PNBP itself is inhibited, rather than activated by, ApoC-II (83, 85).

In the search to identify more physiologically relevant non-lipoprotein substrates, several TG analogs were developed where the lipolytic products could be visualized, and hence LPL activity, quantified by fluorescence (54, 86). Arguably the most sensitive, and well characterized TG-analog, with the most stable lipolytic end products is the EnzChek™ substrate (54). However, the utility of this method to measure LPL activity in human PHP has not been previously demonstrated. Here we show that a 1:40 dilution (in assay buffer) of PHP is sufficient to quantify lipase activity. However, it is important to note that since the EnzChek™ substrate contains a C12 fatty acid side chain in the *sn-1* position, this is subject to hydrolysis by LPL and HL, and therefore nAngptl4-mediated inhibition of LPL is a requisite control. In addition, the inability of ApoC-II to increase LPL activity in HR LPL enriched fractions, when ApoC-II has such a profound ability to increase LPL activity in the triolein-based assay suggests that the interaction between the EnzChek™ substrate, LPL, ApoC-II and Angptl4 needs to be further evaluated. Overall, although questions remain regarding the physiological relevance of the EnzChek™ substrate we recommend this assay in studies using a large volume of biological replicates. Moreover, due to its high-throughput nature this assay can be readily applied to drug discovery screens, but we would also recommend testing promising drug candidates using the triolein-based method to recapitulate key findings.

## Conclusion

In summary, we have shown that including synthetic ApoC-II peptides and nAngptl4 fragments in triolein and EnzChek™-based lipase assays can accurately determine LPL-specific activity using a range of biological samples, such as PHP, HR LPL fractions from LPL secreting cells, and bLPL. We recommend the addition of ApoC-II and nAngptl4 as a cost-effective and reproducible way to measure LPL activity avoiding the variability of human serum or isolated human-VLDL. Moreover, in samples with increased TGs where the LPL activity appears uncompromised, we also advise trialing triolein-based assays using a patient’s serum to determine the relative abundance of factors that may compromise LPL-activity, although further studies will be needed to identify these factors. Finally, in instances requiring high-throughput measurements of LPL activity, we recommend using the EnzChek™ fluorescence substrate, in combination with ApoC-II and nAngptl4 controls to ensure LPL-specific lipase activity is being measured.

## Data Availability Statement

The raw data supporting the conclusions of this article will be made available by the authors, upon request.

## Ethics Statement

The studies involving human participants were reviewed and approved by the Colorado Institutional Review Board. The patients/participants provided their written informed consent to participate in this study.

## Author Contributions

KB and RE contributed to the conceptual design of the study. KB, DO, HW, JM, and EL carried out the experimental studies and data analysis. KB, DO, RE, PR, and KS wrote the manuscript. All authors edited the manuscript.

## Conflict of Interest

The authors declare that the research was conducted in the absence of any commercial or financial relationships that could be construed as a potential conflict of interest.

## Publisher’s Note

All claims expressed in this article are solely those of the authors and do not necessarily represent those of their affiliated organizations, or those of the publisher, the editors and the reviewers. Any product that may be evaluated in this article, or claim that may be made by its manufacturer, is not guaranteed or endorsed by the publisher.

## Abbreviations

Angptl4: Angiopoietin-Like 4
bLPL: bovine LPL
BSA: Bovine Serum Albumin
CVD: cardiovascular disease
CXN: calnexin
EL: endothelial lipase
ER: endoplasmic reticulum
fAngptl4: full-length Angptl4
FFA: free fatty acids
GPIHBP1: glycosylphosphatidylinositol-anchored high-density lipoprotein–binding protein 1
HDX-MS: Hydrogen Deuterium Exchange Mass Spectrometry
HL: hepatic lipase
HR: heparin release
HTG: hypertriglyceridemia
KRP: Krebs Ringer Phosphate
LMF1: lipase maturation factor
LPL: lipoprotein lipase
nAngptl4: N-terminal domain Angptl4
PC: proprotein convertase
PHP: post-heparin plasma
SorLA: sortilin-related receptor with type-A repeats
TG: triglyceride
TRL: TG-rich lipoproteins
VLDL: very-low density lipoproteins.
