# Supplementary material for: Using Synthetic ApoC-II Peptides and nAngptl4 Fragments to Measure Lipoprotein Lipase Activity in Radiometric and Fluorescent Assays
Source: Front Cardiovasc Med. 2022 Jul 14;9:926631. doi: 10.3389/fcvm.2022.926631 (PMC9329559; doi:10.3389/fcvm.2022.926631)
Supplement: Supplementary file 1 [file Data_Sheet_1.docx]

**Supplementary Methods**

**1.EnzChek^TM^ Assay - Statistical analysis**

The fold change of each value from each well per timepoint was added into an XY table in GraphPad Prism V8 for nonlinear fit analysis (**Supplementary Figure 1**). All wells in each condition were then averaged and the standard error calculated. These values were then plotted, and the Michaelis-Menten model in GraphPad was used (**Supplementary Figure 2A-B**). Minimal changes were made to the default settings. Vmax, Km, and the Range were calculated in GraphPad. An analysis was done in GraphPad to ensure the Michaelis-Menten model was appropriate by comparing it to the Allosteric sigmoidal model (**Supplementary Figure 2C**). Normality of the data sets was calculated using the D'Agostino-Pearson normality test in GraphPad (**Supplementary Figure 4D**). Differences were analyzed by two-way ANOVA at each individual time point, and multiple comparison analysis, i.e., comparing each treatment groups (e.g., Angptl4 and ApoC-II) to vehicle control was performed using Dunnett post hoc analysis.


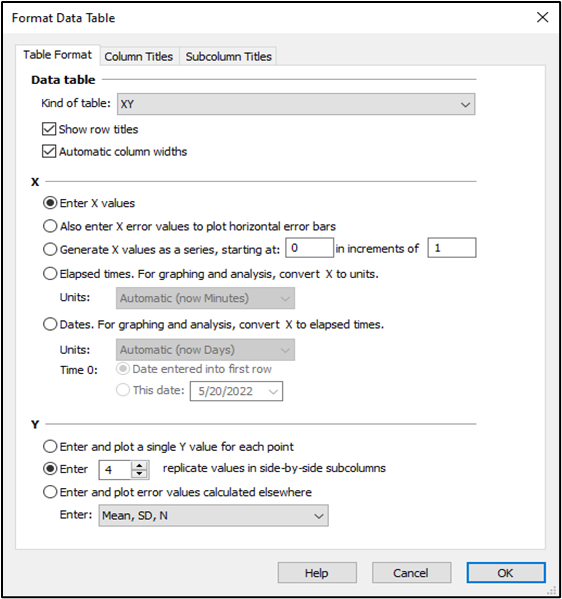


**Figure 1: General statistical formatting for EnzChek™ assay in GraphPad Prism V8**. Data was input in a standard XY Table, wherein timepoints were the X axis and fold change from baseline as the Y axis with each technical replicate being grouped by condition.

**
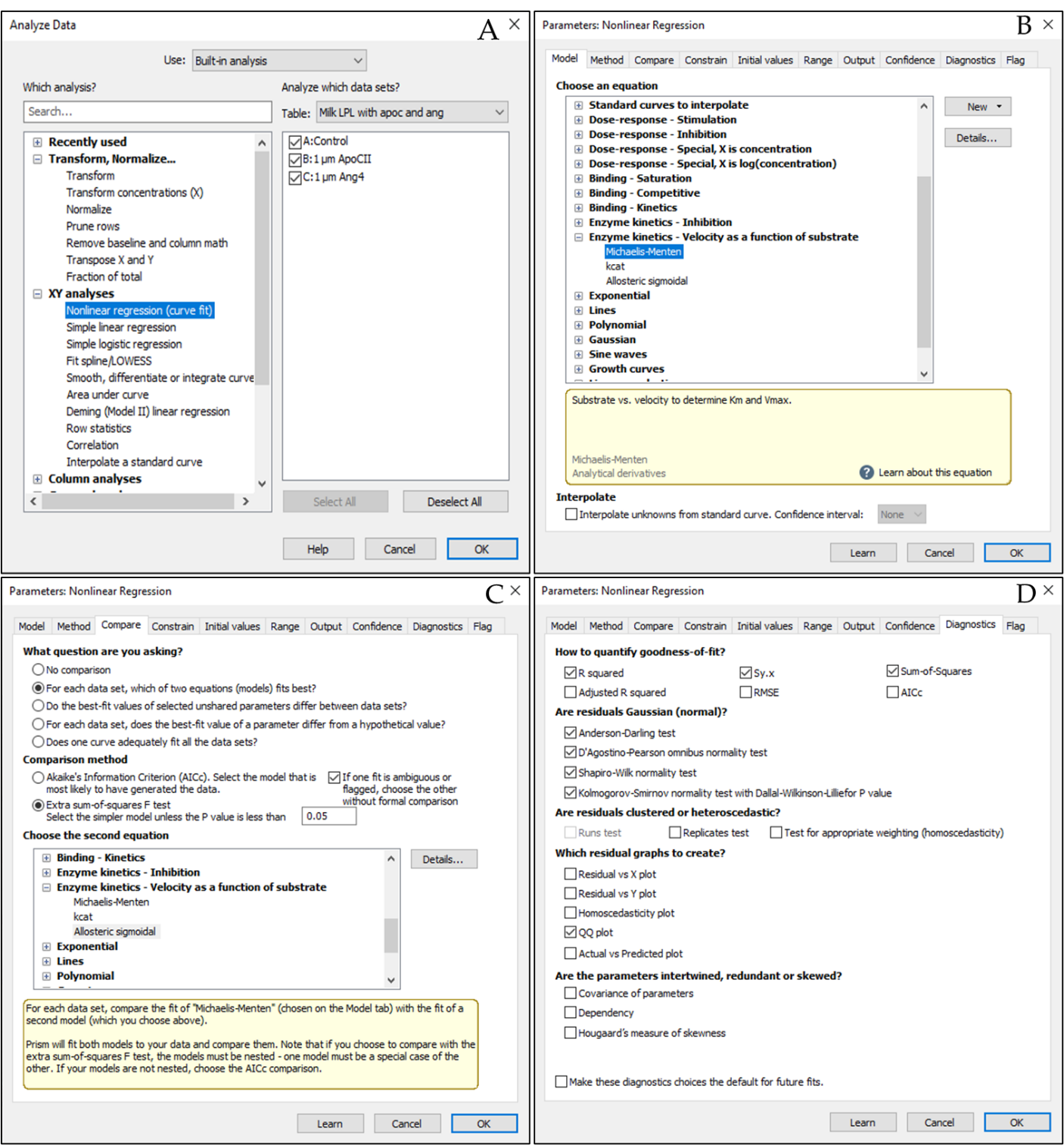
**

**Figure 2: Statistical analysis for EnzChek™ assay in GraphPad Prism V8.** (**A**) Nonlinear regression was performed on the XY table (found in the XY analysis tab) for all conditions. (**B**) The Michaelis-Menten model were chosen, and all settings were kept default except for the “Compare” tab and the “Diagnostics” tab. (**C**) To determine the correct model was being used, a comparison of the Michaelis-Menten model to the Allosteric sigmoidal model was added to the analysis in the “Compare” tab. (**D**) Lastly, to confirm that the assumptions of the two-way ANOVA was not violated, residual testing and a QQ plot were added to the analysis

**2. HDOCK analysis**

The online HDOCK webserver was used to perform rigid-body docking between LPL, ApoC-II, and Angptl4 (73). The PDB files for the three proteins were downloaded from the AlphaFold Protein Structure Database (76, 77, 78). The three AlphaFold structures used were sourced in humans and modified prior to docking. The AlphaFold LPL structure was altered so that it did not include the signal peptide (residues 1-27). The AlphaFold ApoC-II and Angptl4 structures were edited to resemble only the residues of the peptide and N-terminal fragment, respectively. Two separate docking calculations were performed, one between LPL and the nAngptl4 fragment, and one between LPL and the ApoC-II peptide. LPL was used as the input receptor molecule, and the nAngptl4 fragment and ApoC-II peptide as the input ligand molecules. The best-scoring output model for each run was used for visualization in VMD (74). For LPL and the ApoC-II peptide, the best docking score was -229.73. For LPL and the nAngptl4 fragment, the best docking score was -280.43.

**Additional References**

73. Yan Y, Zhang D, Zhou P, Li B, Huang SY. HDOCK: a web server for protein-protein and protein-DNA/RNA docking based on a hybrid strategy. Nucleic Acids Res. 2017;45(W1):W365-W73. Epub 2017/05/19. doi: 10.1093/nar/gkx407. PubMed PMID: 28521030; PMCID: PMC5793843.

74. Humphrey W, Dalke A, Schulten K. VMD: visual molecular dynamics. J Mol Graph. 1996;14(1):33-8, 27-8. Epub 1996/02/01. doi: 10.1016/0263-7855(96)00018-5. PubMed PMID: 8744570.

76. <https://alphafold.ebi.ac.uk/entry/P06858>. Lipoprotein Lipase (Human). AlphaFold structure prediction P06858.

77. <https://alphafold.ebi.ac.uk/entry/P02655>. Apolipoprotein C-II (Human). AlphaFold structure prediction.P02655.

78. <https://alphafold.ebi.ac.uk/entry/Q9Z1P8>. Angiopoietin-related protein 4 (mus musculus). AlphaFold structure prediction.Q9Z1P8.
